# Novel Insight into the Concept of Favorable Combination of Electrodes in High Voltage Supercapacitors: Toward Ultrahigh Volumetric Energy Density and Outstanding Rate Capability

**DOI:** 10.1002/gch2.202100139

**Published:** 2022-01-05

**Authors:** George Elsa, Manavalan Vijayakumar, Rajendran Navaneethan, Mani Karthik

**Affiliations:** ^1^ Centre for Solar Energy Materials International Advanced Research Centre for Powder Metallurgy and New Materials (ARCI) Balapur Hyderabad 500005 India

**Keywords:** carbons, high durability, supercapacitors, volumetric capacitance, volumetric energy density

## Abstract

Most of the biomass‐derived carbon‐based supercapacitors using organic electrolytes exhibit very low energy density due to their low operating potential range between 2.7 and 3.0 V. A novel insight into the concept of the different porous architecture of electrode materials that is employed to extend a device's operating potential up to 3.4 V using TEABF_4_ in acetonitrile, is reported. The combination of two high surface area activated carbons derived from abundant natural resources such as industrial waste cotton and wheat flour as sustainable and green carbon precursors is explored as an economical and efficient supercapacitor carbon electrode. Benefitting from the simultaneous achievement of the higher potential window (3.4 V) with higher volumetric capacitance (101 F cm^–3^), the supercapacitor electrodes exhibit higher volumetric energy density (42.85 Wh L^–1^). Bimodal pore size distribution of carbon with a tuned pore size and high specific surface area of the electrode can promote the fast transport of cations and anions. Hence, it exhibits a high rate capability even at 30 A g^–1^. In addition, the electrodes remain stable during operation cell voltage at 3.4 V upon 15 000 charging–discharging cycles with 90% capacitance retention.

## Graphical abstract

Design and construction of a new configuration using different carbon materials as supercapacitor electrodes can increase the volumetric energy density of supercapacitor device for automobile and other high voltage applications.

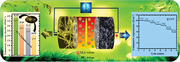

## 1 Introduction

The consumption of electronic equipment's especially portable electronics has drastically increased in the entire globe. Thus, the demand for electrical energy storage devices especially lithium‐ion batteries (LIBs) and supercapacitors (SCs) is essentially increased due to their higher energy density and excellent power delivery, respectively.^[^ ^1^, ^2^, ^3^, ^4^, ^5^, ^6^ ^]^ Supercapacitors or electrochemical capacitors (ECs) showed extensive research attention owing to their superior power density and very long durability.^[^ ^6^, ^7^, ^8^, ^9^, ^10^ ^]^ So far, several carbon materials have been studied as electrode materials in ECs.^[^ ^11^ ^]^ However, poor volumetric capacitance <50 F cm^–3^ and volumetric energy density <20 Wh L^–1^ of different biomass‐derived carbon are major obstacles for commercial applications because of their low packing density of electrodes (≈0.6 g cm^–3^), which extends to the size of fabricated ECs, particularly for electric vehicle (EV) applications.^[^ ^12^, ^13^ ^]^ In addition, the perplexed fabrication process and spectacular price of the device further hindered the upscaling of the materials for commercialization volumetric energy density. The low volumetric capacitance (50–80 F cm^–3^), energy (20–30 Wh L^–1^), and inferior rate capability are the drawbacks bound to the commercial activated carbon (AC).^[^ ^1^, ^5^, ^14^ ^]^ Therefore, the exploitation of cost‐effective carbon materials with improved volumetric energy density and excellent rate capability needs to encounter the requirement of the portable devices and EVs that required high voltage window.

From a long‐term perspective, biomass‐derived carbon can embellish or supersede commercial carbon, by tendering a great value for next‐generation electrode materials from biomass.^[^ ^1^, ^2^, ^15^, ^16^, ^17^ ^]^ The porous AC materials derived from different biomass showed remarkable electrochemical functioning as an individual characteristic of material such as high gravimetric capacitance, good cycling stability, high packing density, structural morphology, and improved rate capability.^[^ ^5^, ^18^, ^19^ ^]^ It was encountered that the “positive” electrode has a capacitance greater than the “negative” electrode, and the divergence among the “positive” and “negative” electrodes in LiPF_6_ in PC is more prominent than TEABF_4_ in PC electrolyte. As well, the narrow operating potential range at “positive” and “negative” voltage seems to be asymmetric in both electrolytes.^[^ ^5^, ^8^ ^]^

With an ever‐increasing demand for portable electronics and hybrid EVs, SCs have shown more attention due to its characteristics such as greater power density and durability. In SCs, ACs derived from coconut shells are commercially used as both positive and negative electrodes.^[^ ^15^, ^16^ ^]^ However, there is an issue with voltage stability when they are employed in high voltage and high‐temperature applications such as automobiles since the operating potential of an individual ACs device is confined to the potential of 2.7 V and more such devices should be stacked in multiple numbers to get the demanded potential (ordinarily 50 to 100 V for electric two‐wheelers and 300 to 500 V for electric four‐wheelers and electric buses). Hence, improving the potential of a single device can be a possible solution to reduce the number of cells for stacking as well as in creating a compact module. The increase in the potential of a single cell not only contributes to an overall voltage of the stacked device but also in increasing the overall energy of the device (*E* = ½ CV^2^). Hence, extending the potential (*V*) of the cell can have a huge enhancement on the overall device energy.

In the present contribution, we describe a novel insight into the concept of the type of electrode materials (positive and negative) that can be used to extend the potential of the supercapacitor cell up to 3.4 V using TEABF_4_/acetonitrile organic electrolyte. For the SCs device, two different carbon‐based materials were used as electrode active materials based on the concept of pore size engineering of materials according to ionic sizes of solvated ions in the electrolyte. As we know that “+” (tetraethylammonium) and “−” (tetrafluoroborate) ions of the electrolyte salt are of different sizes: bare TEA^+^ is 0.68 nm and BF_4_^–^ is 0.48 nm, whereas, after solvation, the sizes are further increased (TEA^+^ is 1.30 nm/BF_4_^–^ is 1.16 nm).^[^ ^20^ ^]^ Hence it is clear that since, both the ionic sizes of electrolyte salts are being dissimilar, it would be appropriate to use electrode materials that are fine‐tuned accordingly to accommodate the different size ions. Thus, we have designed a novel asymmetric SC using wheat‐derived carbon as “+” electrode and cotton‐derived carbon as “−” electrode, where fine‐tuned electrodes accommodate the bulky ions, thus extending the overall device operating window. It is feasible to extend the overall electrochemical stability and voltage of the device by this asymmetric configuration, where the 3.4 V potential makes it possible to accomplish greater energy density than conventional ACS twice as comparatively into SCs. Our high‐voltage SC may be suitable candidate for automobiles and other high voltage applications because of its high potential window.^[^ ^21^, ^22^ ^]^

## 2 Experimental Section

### 2.1 Synthesis of Activated Carbon

**Figure 1:**
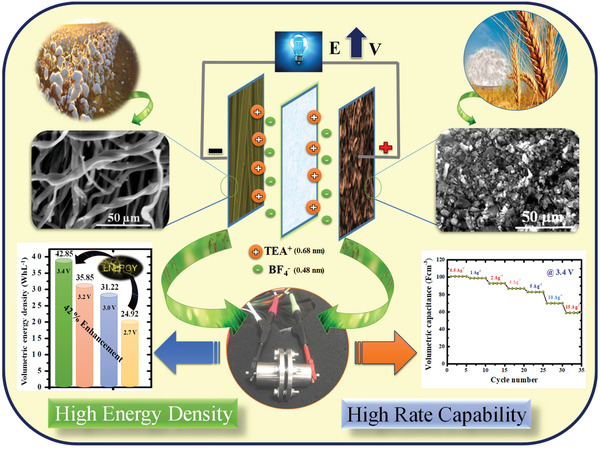
Overall concept of favorable combination of electrodes for high voltage supercapacitors.

Two dissimilar biomass samples such as wheat flour and waste cotton were converted into AC by the processes of carbonization and followed by activation. Initially, wheat flour and waste cotton were calcined under nitrogen atmosphere at 600 °C for 1 h and the carbon yield from wheat flour and cotton were calculated around 25 and 20 wt%, respectively. After carbonization, the chemical activation was carried out by using an activation agent of potassium hydroxide (KOH). In typical chemical activation, the carbon and KOH were thoroughly mixed in a ratio of 1:3, and the carbon samples were activated at 850 °C for 1 h with 5 °C min^–1^ as a ramping rate under the N_2_ atm. After KOH chemical activation, the obtained AC samples were washed with a solution of 1 m HCl separately till the pH reached 7.0, and then, the AC samples were dried at 100 °C for 12 h. The AC samples derived from wheat flour and waste cotton were denoted as activated wheat flour (AWF) and activated carbon fiber (ACF), respectively in the entire article. The concept of sustainable large‐scale fabrication of two different micro‐mesoporous ACs derived from wheat flour and waste cotton with high volumetric energy density is illustrated in **Figure** **1**.

### 2.2 Characterization of the Samples

The carbon samples were characterized by using field emission‐scanning electron microscopy (FE‐SEM) with a cross‐section of the electrodes on the carbon‐coated aluminum current collector (Zeiss Gemini 500). The amorphous nature of ACF and AWF were analyzed by using XRD (Bruker analytical X‐ray systems D8 advanced system) and Raman spectrometer (Horiba Jobin Yvon Lab Ram HR‐800). The specific surface area, pore‐volume, and pore size of the carbon samples were measured by using N_2_ adsorption and desorption analysis (Brunauer–Emmett–Teller, Micromeritics, ASAP 2020). The micropore size distributions of the ACF and AWF were determined by using 2D‐non‐local density functional theory (2D‐NLDFT).^[^ ^23^, ^24^ ^]^

### 2.3 Electrode Preparation

The “positive” and “negative” electrodes for the SC were fabricated by mixing of 80 wt% of active material (activated carbon), 10 wt% of binder (polytetrafluoroethylene (PTFE), (C_2_F_4_)_*n*_), and 10 wt% of conductive carbon black as a conductive additive. The ratio was maintained for making the positive and negative SC electrodes made from ACs derived from wheat flour (AWF) and waste cotton (ACF), respectively. The above mixture was uniformly mixed and then the carbon dough was prepared by the addition of a few drops of ethanol. The two flexible and free‐standing carbon electrode strips were prepared by the rolling method. The flexible carbon strips were pressed on carbon‐coated aluminum foil as a current collector by using a hot roller pressing machine to obtain a dense supercapacitor electrode with controlled thickness. Then, the hot‐pressed electrodes were dried at 100 °C for 12 h. The SC electrodes with 12 mm in diameter and 150 ± 10 µm (ACF) // 110 ± 10 µm (AWF) of thickness were prepared and the active mass of each electrode was controlled at around 12 ± 1 mg cm^–2^. The coin cell assembly (2032‐type coin cell) was used to fabricate the SC cells. 1 m tetraethylammonium tetrafluoroborate salt in acetonitrile (1 m TEABF_4_/AN) was used as an organic electrolyte. The coin cells were fabricated in a glove box under an argon (Ar) atmosphere.

The electrochemical characteristics of SC cell with two different carbon electrodes with two‐electrode configuration were tested and the detailed formulas and equations for the calculation of specific capacitance, energy density, and power density were extensively reported by our group.^[^ ^21^, ^22^, ^25^, ^26^ ^]^

## 3 Results and Discussion

### 3.1 Characterization of the Samples

**Figure 2:**
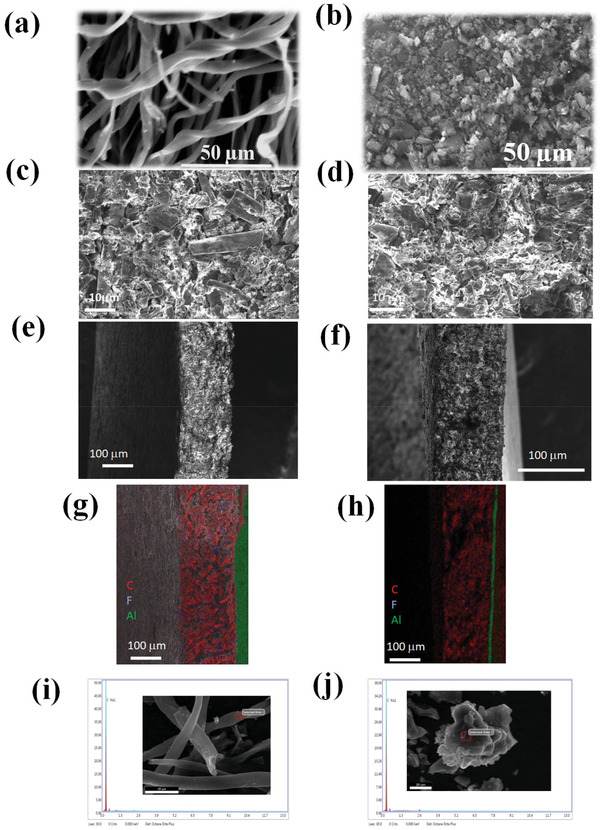
Surface morphology of the supercapacitor carbons examined by SEM analysis: a) ACF, b) AWF, c) ACF electrode, d) AWF electrode, e,f) cross‐section of ACF and AWF electrodes, g,h) elemental mapping of cross‐section of ACF and AWF electrodes, i,j) EDAX analysis of ACF and AWF.

The morphology of the AWF and ACF was analyzed by using SEM as shown in **Figure** **2**. It is very interesting to observe that the two different carbon precursors were having two different morphological characteristics such as the fiber morphology from the waste cotton fibers and very dense particle morphology from wheat‐derived carbon. The waste cotton fibers are turned into thin carbon fibers upon carbonization at 600 °C for 1 h and followed by KOH chemical activation at 850 °C for 1 h with the carbon fiber average diameter of 8–10 μm. On the other hand, the wheat flour sample after carbonization and activation with KOH showed high dense particle morphology with uniform particle sizes of around 5–10 μm as depicted in Figure 2a–d. The SC electrodes packing density was very important for the volumetric capacitance of the SC.^[^ ^27^ ^]^ Thus, the cross‐section view of the two carbon electrodes of AWF and ACF was examined. It could be visualized from SEM images that both electrodes exhibited high material adhesion to the carbon‐coated current collector (Figure 2e–h). The cross‐section view of both microstructures of the electrodes clearly showed the high packing density of both electrodes. It was observed from the SEM images that there was a strong adhesion between the carbon material and carbon‐coated aluminum current collector where the current collector used was carbon‐coated aluminum foil. Aluminum foil was deposited with a few micrometers thick carbon deposits to reduce the contact resistance between the carbon electrode and the current collector which was the influencing parameter in high current rates. At a constant mass of 12 ± 1 mg cm^–2^ per electrode, the supercapacitor electrode thickness of the AWF was found to be around 110 ± 10 µm and the thickness of the ACF was found to be 150 ± 10 µm. It was observed that the ACF electrode thickness was greater than that of the AWF electrode because of its flappy and fibrous nature ACF. The packing density of AWF and ACF electrodes were 0.76 and 0.70 g cm^–3^, respectively. The packing density of the electrode could play a vital role in attaining a higher volumetric energy density of the SC.^[^ ^27^ ^]^

The elemental compositions of the AWF and ACF were analyzed by using EDAX (Figure 2i–j). From the EDAX, it was confirmed that the two samples contained only one element that was carbon only and no other impurities in it and it was the necessity for carbon–carbon‐based SC application. ACF electrodes with a highly microporous structure and macroporous interconnected fiber network could provide a good electrical conductivity with an excellent pathway for the electrolyte diffusion that helped in fast ion transport which increased the specific capacitance of the electrode.^[^ ^21^, ^26^, ^28^ ^]^ On the other hand, AWF electrode with highly dense packed particles gave rise to high packing density for the electrode which significantly impacted the electrode volumetric capacitance. Thus, the combination of positive and negative electrodes was highly suitable for researching greater power and energy density to enhance the overall capacitive performance. Furthermore, the amorphous nature of the two different activated carbons (AWF and ACF) was confirmed by XRD and Raman spectroscopic techniques as depicted in Figure S1 in the Supporting Information.

**Figure 3:**
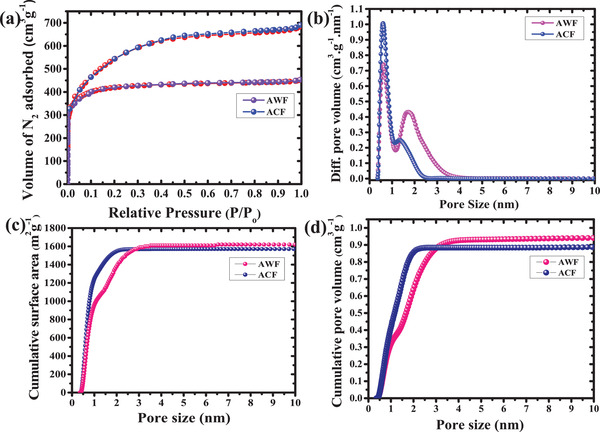
N_2_ adsorption–desorption isotherm of ACF and AWF: a) BET isotherm, b) average pore size versus pore volume, c) cumulative surface area versus pore size, d) cumulative pore volume versus pore size.

N_2_ adsorption and desorption measurements of AWF and ACF are depicted in **Figure** **3**a–d. The isotherms of both carbon materials are having type I and IV isotherms which represented the availability of the distinctive micropores and mesopores in the carbon material as illustrated in Figure 3a which showed the combination of micropores and mesopores in the samples.^[^ ^20^, ^29^ ^]^ The 2D‐non‐local density functional theory (2D‐NLDFT) of the carbon sample with micropore size distribution is represented in Figure 3b which indicates the presence of bi‐modal pore size distribution between 0.6 to 0.9 nm and 1.3 to 2.8 nm for AWF and 0.7 and 1.3 nm for ACF, respectively. The specific surface area of the activated carbon derived from AWF and ACF was around 1620 and 1550 m^2^ g^–1^, respectively. In addition, the cumulative surface area versus pore size of both activated carbons derived from 2D‐NLDFT is shown in Figure 3c. From Figure 3d, the cumulative pore volume versus pore size of the two different activated samples (ACF and AWF) was around 1.35 and 0.97 cm^3^ g^–1^ respectively. The presence of the mesoporous structure was responsible for fast ion transport and the microporous structure corresponded to the effective electric double‐layer capacitor (EDLC) layer formation. For commercial application, the optimum textural characteristic was the pre‐requisite which was offered by the present porous carbon which was having a bi‐modal structure that contributed to high gravimetric and volumetric performance.^[^ ^18^, ^30^, ^31^ ^]^

Since cotton was made of cellulose fiber and the activated carbon derived from waste cotton (ACF) also possessed a highly microporous structure obtained by KOH activation and the interconnected macroporous fiber network which could provide a very good pathway not only for the electrolyte diffusion but also electrolyte ion transfer. Besides, the interconnected macroporous fiber network could also provide uniform and good electrical conductivity throughout the supercapacitor electrode. The carbon fibers could improve the mechanical stability of the electrode layer as well as provide long‐range electrical conductivity. On the other hand, the activated carbon derived from wheat flour had a unique porous structure with bi‐modal pore size distribution. After KOH activation, the surface of the carbon particles exhibited crumpled sheet‐type morphology. The wrinkled morphology of the carbon nanosheets could provide higher accessible surface area and interconnected porous network which could favor the electrode–electrolyte interactions. Besides, the nanosheets structure could substantially accelerate the transfer of the electron that could boost up the rate performance.

### 3.2 Electrochemical Performance of ACF and AWF Electrodes

To evaluate the combined properties of two different electrodes, the two‐electrode configuration is fabricated by using 1 m TEABF_4_/AN (0–3.4 V) as an organic electrolyte. The mass of the SC electrode should have 8–12 mg cm^–2^ (active mass) with a thickness of 100–250 μm for commercial application.^[^ ^7^, ^26^, ^32^ ^]^ The high active mass loading per cm^2^ is invariably an advantage since it enhances the volumetric energy density which increases the compactness of the device and also decreases the manufacturing cost for the fabrication of electrodes. By considering the above factors, the effects on combination properties of AWF's high‐density electrode on one side with a high gravimetric capacitance electrode from the fiber nature of the ACF electrode on the other side are necessary to investigate.

#### 3.2.1 Effects of Individual and Combined Properties of The Two Different Morphological Carbon

The concept of the symmetric configuration of the same morphological material SCs on two carbon sources ACF and commercial carbon YP‐50F were studied and reported by our group^[^ ^33^ ^]^ using polyvinylidene difluoride (PVDF) as a binder, in that study a very poor rate capability was observed because of the distortion cyclic voltammetry (CV) curves in the symmetry system while enhancing the operating potential up to 3.0 V in an organic electrolyte. From the above discussion, before going for the two different morphological combinations of the carbon electrodes in symmetry SC configuration it is necessary to evaluate the same morphological carbon ACF and AWF electrodes using PTFE binder in symmetry configuration. The symmetric cells were fabricated and investigated in an organic electrolyte by using 1 m TEABF_4_/AN.

**Figure 4:**
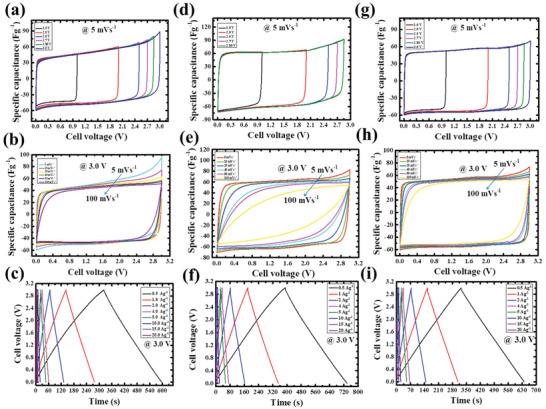
Electrochemical performances of ACF, AWF and combination of supercapacitor electrodes: a–c) ACF, d–f) AWF, g–i) combination of supercapacitor electrodes.

The detailed CV profiles of symmetric configurations of the same and a combination of ACF and AWF SC electrodes were shown in **Figure** **4**a–c. At 5 mV s^–1^ constant scan rate of the CV curves with an enlarged potential window up to 3.0 V for the three symmetry systems. It is observed that symmetric configuration of same morphological carbon electrode showed a quasi‐rectangular shape at a voltage of 2.85 to 3.0 V. But the different morphological carbon in symmetry system exhibited a shape of perfect rectangular which indicates proof of ideal SC characteristic. It is observed that the symmetry system of two different morphological materials showed a better rate capability than the similar morphological materials as represented in Figure 4d–f. From the observation, it can be concluded that the symmetric configuration of different morphological carbon electrodes indicated a superior SC performance and improved rate capability as compared with the symmetric configuration of the same morphological carbon electrodes. However, one can see from Figure 4d–f that CV curves of the symmetric configuration of cotton show good rate capability at high and medium scan rates but it is observed that at low scan rates there is a pore restriction at 3.0 V and from the wheat symmetry system also shows poor rate capability profile but it shows the advantage of having rectangular shape at lower scan rates. From the above observation, the symmetry configuration of the two morphological electrodes showed a better rate capability and stable potential window at 3.0 V. It is evidence that the symmetry system of different materials utilizes the unique properties of the materials in the SC system and it increases the performance of the SC.

The result is furthermore confined with the galvanostatic charge‐discharge (GCD) graphs and depicted in Figure 4g–i. In this case, the two identical morphological positive and negative electrodes of respective ACF and AWF individuals behave the same when compared with the composite system of “+” and “–” electrodes for SC. The charging and discharging profile shows a perfect triangular shape and columbic efficiency is around 99% for the composite system at low current densities but the same morphological carbon system shows columbic efficiency of 94% at a low current density which shows no room to store the charge at a higher voltage for the same morphological carbon system. From the above discussion, it is possible to obtain a higher voltage by choosing different materials as positive and negative electrodes for the symmetry SC system. In this case, the AWF electrode has high areal capacitance. From the above outcomes, it can be resolved that composite carbon electrodes with AWF as negative electrodes exhibited a higher charge storage medium and consequently it has higher volumetric energy density.

**Figure 5:**
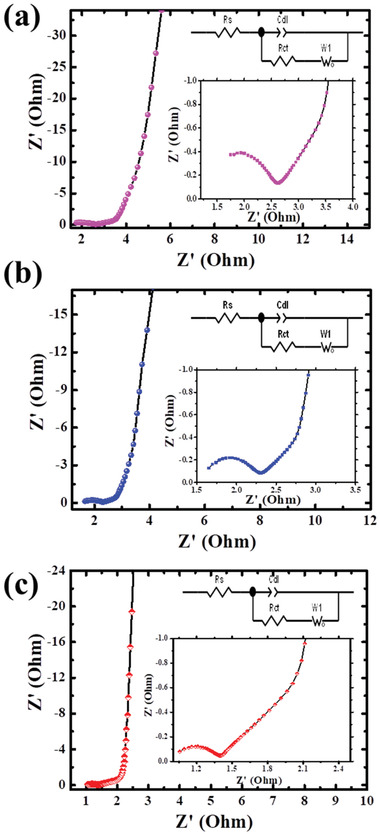
EIS of supercapacitor electrodes: a) ACF, b) AWF, c) combination of ACF//AWF supercapacitor electrodes.

The electrochemical performance of SCs is further compared and confirmed by the EIS technique. In general, the EIS was studied between the frequency range from 0.01 Hz to 100 kHz to find the charge transfer and series resistance of the electrode.^[^ ^34^, ^35^ ^]^ Furthermore, all the SC electrodes follow the same fabrication setup, and hence an identical value of contact resistance is obtained for all the fabricated SC cells. Besides, symmetric composite electrodes revealed a low series and low ionic resistance than symmetric ACF and AWF electrodes as depicted in **Figure** **5**a–c. Here we need to mention that the resistance of all the symmetric configuration system electrodes showed very low. It may be attributed to comfortable ionic diffusivity within the two electrodes (positive and negative) of all the symmetric configurations without creating any ionic residence at the low frequency to the mid‐frequency region. But it is noticed that the composite symmetric configuration cell shows highly conductive and it is vertical to the Nyquist plot's imaginary axis in comparison with the other two same morphological symmetry systems at the high‐frequency region, Nyquist plot with a slight inclination at the mid‐low frequency region was noticed because of its same “+” and “–” electrode material.

**Figure 6:**
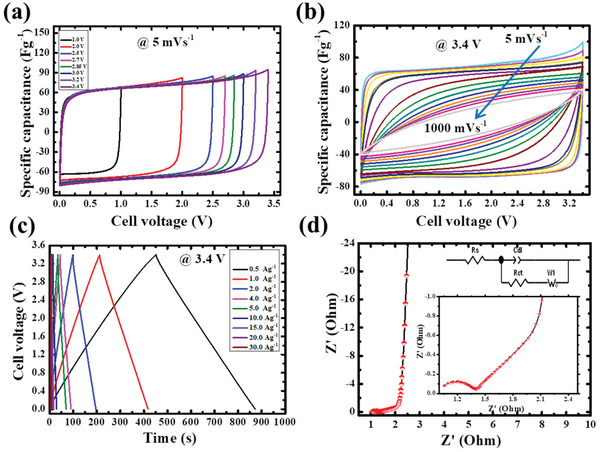
Electrochemical performances of combination of ACF//AWF supercapacitor electrodes. a) CV curves at 5 mV s^–1^, b) CV curves with different scan rates (5–1000 mV s^–1^), c) GCD, d) EIS.

To verify potential enlargement of SC, the composite electrode was tested at an extended operating window from 3.0 to 3.4 V and the electrochemical tests were carried out at 3.4 V. **Figure** **6**a depicts the 5 mV s^–1^ CV profile up to 3.4 V shows the rectangular shape which indicates the ideal capacitor even at very high potential. And also, Figure 6b shows the CV curves from 5 to 1000 mV s^–1^ at 3.4 V showed the high rate capability of the composite SC device. The GCD graph between 0.5 and 30 A g^–1^ of current density are represented in Figure 6c. When combining two different morphological materials the tuning parameter for the electrodes is reduced in the long strip process for the higher farad cells and also operating voltage of the cell is increased as compared to sample morphological SC cell fabrication. The performance of the composite electrode showed higher capacitance and higher potential window as compared with the same morphological symmetry system (Table S1, Supporting Information). It is observed that one electrode has a high packing density and another electrode has a high specific capacitance which enhances the overall gravimetric and volumetric capacitance of the cell. It is challenging to achieve 3.4 V in organic electrolytes. Recently the operating voltage of the commercial Maxwell SC was enlarged to 3.0 from 2.85 V. The main advantage of increasing the operating potential is to enhance the SC cell energy density, for example, 3400 F cell with 2.85 V has an energy density of around 7.4 Wh kg^–1^ and now the same configured cell with the increased voltage of 3.0 V is having the energy density of 8.57 Wh kg^–1^ with same cell configuration,^[^ ^22^ ^]^ respectively. However, in the present study, the smart combination of the two dissimilar composite carbon electrodes ACF and AWF can increase the cell voltage and high volumetric capacitance which can reduce the overall size of the SC device. The main finding of the current article is to underscore the improved performance of EC with composite carbon‐based electrodes in an organic electrolyte at 3.4 V. It can be observed from Figure 6d that EIS characteristics of the composite symmetric system show less ESR and are consistent with GCD. Furthermore, the Nyquist plot shows that cell is highly conductive in the region of low‐frequency.

**Figure 7:**
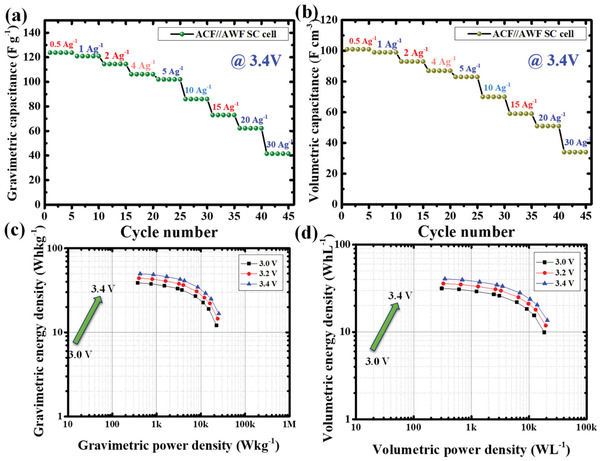
The rate capability, energy and power densities of combination of ACF//AWF supercapacitor electrodes. a) Gravimetric capacitance at various current densities, b) volumetric capacitance at various current densities, c) gravimetric energy density versus power density, d) volumetric energy density versus power density.

The gravimetric and volumetric capacitance at different current densities is shown in **Figure** **7**a,b. It is noticed that the composite electrode is showed a high gravimetric capacitance of 125 F g^–1^ @ 0.5 A g^–1^ at 3.4 V. The values of the volumetric capacitance are depending on the packing density of the electrode as well as the gravimetric capacitance of the electrode. High gravimetric capacitance and the high electrode density of the AWF can contribute to the high volumetric capacitances. The combined property of the two electrodes (AWF//ACF) in a single system showed higher volumetric capacitance of 101 F cm^–3^ at 0.5 A g^–1^ at 3.4 V. The excellent performance of carbon composite electrodes could be attributed to the combination of ACF's unique 3D interconnected network structure and AWF's high particle density electrode with narrow pores for the rapid ion transport within the pores of the electrodes which promotes higher cell voltage. To reduce the surface contact resistance in an SC cell, carbon‐coated aluminum foil for the enhanced conductivity has been used, and eventually, it reduces the surface to contact resistance value considerably.^[^ ^36^ ^]^

The gravimetric/volumetric energy density of the supercapacitor is represented in Figure 7c,d. The energy density increases with the increase in the voltage of the cell. The voltage increment indicates that the composite SC cell has promising high gravimetric and high volumetric energy density at 3.4 V. The energy density increases with increasing the voltage as per the equation *E* = ½ CV^2^. And also with the high dense electrode on the one side has more space for ion storage at the device level. The volumetric energy density of composite electrodes at 3.4 V using 1 m TEABF_4_/AN showed a higher value than other SC biomass electrodes.

**Figure 8:**
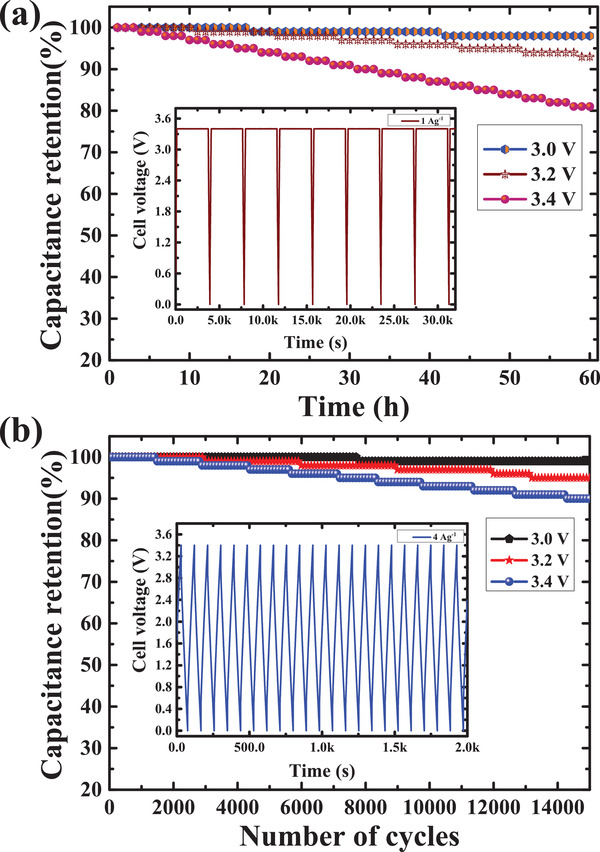
Durability test of the supercapacitor electrodes. a) Cycling stability, b) holding test.

To examine the EC's stability, the cycling tests, and aging tests were carried out for the composite symmetry SCs.^[^ ^37^, ^38^ ^]^ The voltage holding (aging/floating) test was executed by holding the capacitor at a rated operating potential for 1 h then the value was measured upon specific GCD cycles with the function of time. The time versus capacitance retention is illustrated in **Figure** **8**a. In an aging test, the EC cell was charged up to 3.0, 3.2, and 3.4 V at 1 A g^–1,^ and the representing higher potential was held for 1 h constantly. Then, the cell was allowed to the discharge voltage of 0 V at 1 A g^–1^. The above cycles were repeated for 60 h. It was found that capacitance degradation happens after 60 h of the cell at 3.0, 3.2, and 3.4 V and it is around 98%, 92%, and 80%, respectively. It shows that cells have less capacitance degradation after 60 h at 3.4 V. However, there is not much variation in the degradation of the capacitance for the cell tested on normal GCD test at 3.0 to 3.4 V as illustrated in Figure 8b. The results confirm the importance of the aging test rather than the normal cycling tests for testing the cyclic performance of the SC at a limit of higher potential. Usually, the cycling test consumes more time compared to the floating test but it is not so critical experiment to assess the durability of the supercapacitor cell.

## 4 Conclusions

The synthesis of AC derived from two different morphological biomass precursors namely wheat flour and industrial waste cotton as carbon resources for high volumetric energy density SC electrodes with a high operating voltage of 3.4 V were successfully demonstrated. Besides the SC development with the same morphological carbon electrodes, an effort has been drawn to combine two different morphological carbon electrodes in a single SC cell for the combined properties of the individual performance of each carbon for the overall cell performance of SC. The voltage‐holding and cycling tests were carried out and their electrochemical performances were examined. It was found that it is feasible to extend the overall electrochemical stability and voltage of the device by using this asymmetric configuration, where the 3.4 V operating voltage makes two times increased energy density compared with other reported conventional ACs. We strongly believe that the design and construction of a new configuration using different carbon materials as supercapacitor electrodes can increase the volumetric energy density of SCs for automobile and other high voltage applications.

## Conflict of Interest

The authors declare no conflict of interest.

## Supporting information

Supporting Information

## Data Availability

The data that support the findings of this study are available from the corresponding author upon reasonable request.
