# Supplementary material for: Novel Insight into the Concept of Favorable Combination of Electrodes in High Voltage Supercapacitors: Toward Ultrahigh Volumetric Energy Density and Outstanding Rate Capability
Source: Glob Chall. 2022 Jan 5;6(4):2100139. doi: 10.1002/gch2.202100139 (PMC8995712; doi:10.1002/gch2.202100139)
Supplement: Supplementary file 1 — Supporting Information [file GCH2-6-2100139-s001.pdf]

Global Challenges Open Access

Supporting Information

for *Global Challenges,* DOI: 10.1002/gch2.202100139

Novel Insight into the Concept of Favorable Combination of Electrodes in High Voltage Supercapacitors: Toward Ultrahigh Volumetric Energy Density and Outstanding Rate Capability

*George Elsa, Manavalan Vijayakumar, Rajendran Navaneethan, and Mani Karthik**

Novel insight into the concept of favourable combination of electrodes in high voltage supercapacitors: Towards ultrahigh volumetric energy density and outstanding rate capability

George Elsa, Manavalan Vijayakumar, Rajendran Navaneethan and Mani Karthik*

Centre for Solar Energy Materials, International Advanced Research Centre for Powder Metallurgy and New Materials (ARCI), Balapur, Hyderabad-500005, India.

*Email: mkarthik@project.arci.res.in, karthik_annauni@yahoo.co.in

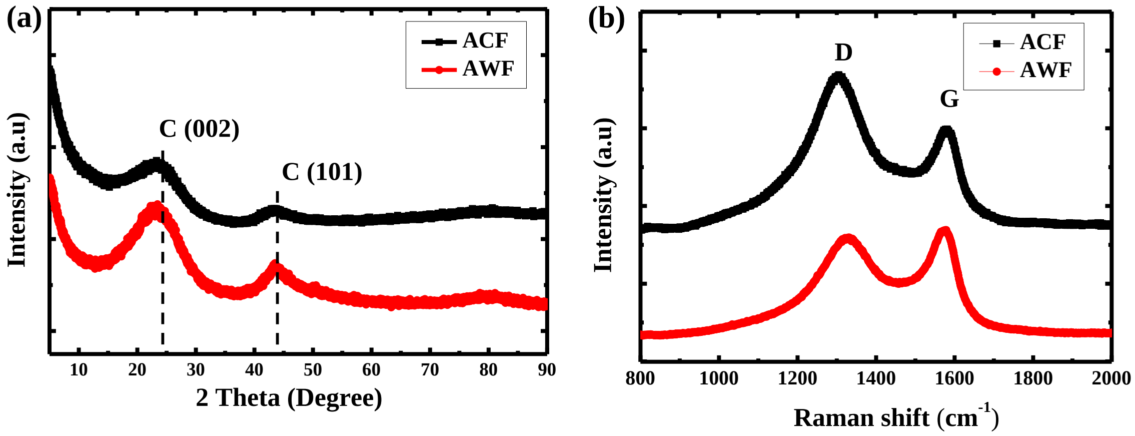

Fig. S1. Structural features of the two different activated carbons (AWF and ACF): (a) XRD pattern, (b) Raman spectrum

The structural features of the two different activated carbons (ACF and AWF) were analysed by using XRD and Raman techniques. The disordered characteristics of the two different amorphous carbon materials were reflected in the XRD and Raman spectrum. From the XRD pattern, the two broad peaks were identified at $2\theta = 25^o$ and $2\theta = 44^o$, which are indexed as (002) and (101) reflections of amorphous carbon material (Fig. S1(a)). On the other hand,

Raman spectroscopy is used to identify the microstructure of the activated carbon material. It is seen from Fig. S1(b) that the two intense peaks have appeared at 1345 cm^{-1} and 1593 cm^{-1} in the Raman spectrum which is assigned to the D band and G band, respectively. It is observed from Fig. S1(b) that ACF has a more amorphous nature but AWF is having a slight graphitic nature. The presence of disordered amorphous carbon is identified in the D band and the G band is usually corresponding to graphite in-plane vibrations.

Table S1 Comparison with other biomass derived carbon-carbon based supercapacitor electrodes reported in the literature

S. No	Biomass	Electrolyte	Voltage (V)	Gravimetric Capacitance (F g^{-1})	Volumetric Capacitance (F cm^{-3})	Current Density (A g^{-1})	Energy density (Wh Kg^{-1})	Power density (KW Kg^{-1})	References
1	Vinasse	1M $TEABF_4$/AC	3	164	-	1	22.7	10.8	[1]
2	Sucrose and Zinc chloride	1M $TEABF_4$/AC	3	130	-	1	55	2.5	[2]
3	Salvia miltiorrhiz a	1M $TEABF_4$/AN	3	130	-	0.5	22	0.448	[3]
4	Waste cotton	1M $TEABF_4$/AN	3.2	-	87	1	30.94	-	[4]
5	Pomelo Peels	1M $TEABF_4$/AN	3	163	-	0.5	50.95	0.44	[5]
6	Cork	1M $TEABF_4$/AN	3	133	-	1	42	0.75	[6]
7	AWF//AC F	1M $TEABF_4$/AN	3.4	125	101	0.5	42.85 Wh L^{-1}	-	Present work

Reference:

[1] C. Shi, L. Hu, K. Guo, H. Li, T. Zhai, Adv. Sustain. Syst. **2017**, 1, 1600011

[2] L. Zhang, Y. Zhu, W. Zhao, L. Zhang, X. Ye, J. J. Feng, J. Electroanal. Chem. **2018**, 818, 51-57.

[3] Y.L. Zhang, Z.S. Tang, Waste Manag. **2020**, 106, 250–260.

[4] M. Vijayakumar, A. BharathiSankar, D. Sri Rohita, T.N. Rao, M. Karthik, ACS Sustain. Chem. Eng. **2019**, 7, 17175–17185.

[5] F. Sun, L. Wang, Y. Peng, J. Gao, X. Pi, Z. Qu, G. Zhao, Y. Qin, Appl. Surf. Sci. **2018**,436, 486–494.

[6] T. Mitravinda, S. Anandan, C.S. Sharma, T.N. Rao, J. Energy Storage. **2021**, 34, 102017.
